# Understanding user perspectives of and preferences for oral PrEP for HIV prevention in the context of intervention scale‐up: a synthesis of evidence from sub‐Saharan Africa

**DOI:** 10.1002/jia2.25306

**Published:** 2019-07-22

**Authors:** Robyn Eakle, Peter Weatherburn, Adam Bourne

**Affiliations:** ^1^ Department of Global Health and Development London School of Hygiene and Tropical Medicine London United Kingdom; ^2^ Sigma Research Department of Public Health, Environments and Society London School of Hygiene and Tropical Medicine London United Kingdom; ^3^ Australian Research Centre in Sex, Health & Society La Trobe University Melbourne Australia

**Keywords:** HIV prevention, pre‐exposure prophylaxis, acceptability, end‐user, public health, key and vulnerable populations

## Abstract

**Introduction:**

Oral pre‐exposure prophylaxis (PrEP) for HIV prevention has been proven to significantly reduce new HIV infections yet scale‐up has been slow. As contexts continue to adjust to make space for PrEP, it is crucial to understand the perspectives and experiences of potential end‐users. In order to inform PrEP and demand creation interventions, this paper examines personal perspectives on adopting and using PrEP among HIV at‐risk populations in sub‐Saharan Africa.

**Methods:**

Using the principles of a scoping review in July 2018, we explored the extent, range, and nature of published literature regarding PrEP uptake and use among; men who have sex with men, HIV serodiscordant couples, adolescent girls and young women, pregnant and breastfeeding women, women partners of migrant workers; and people who use drugs. Steps included: identification of the research question; identification of relevant studies; study selection; charting the data; and collation – summarizing and reporting results. PubMed and PsycInfo were searched for papers relating to PrEP uptake and use in sub‐Saharan Africa. Resulting papers were reviewed with data extracted and compiled in Excel for analysis. A broad content analysis was conducted and organized into high‐level themes.

**Results and discussion:**

Thirty‐five papers were included in this review. There was little opposition in general to oral PrEP; however, there were significant nuances in its broader acceptability, applicability, and usability. We identified five themes within which these are discussed. These relate to balancing complexities of personal empowerment and stigma; navigating complex risk environments; influences of relationships and partners; efficacy and side effects; and practicalities of use. This body of research suggests that while product attributes and the logistics of PrEP delivery and use are important topics, it is vital to consider stigma, the interactions of PrEP use with relationships, and the need for broader understanding of ARVs for prevention versus treatment.

**Conclusions:**

Planning for, programming and promoting the adoption of oral PrEP necessitates a deeper understanding of end‐user priorities in order to ensure successful interventions. This review illustrates the nuances facilitating or deterring PrEP use that may affect the larger effort of PrEP scale‐up.

## 1 Introduction

Oral pre‐exposure prophylaxis (PrEP) for HIV prevention has been proven to significantly reduce new HIV infections in efficacy trials 1, yet introduction has been slow. PrEP is being implemented in nearly 50 countries to varying degrees 2, though predominantly in phased implementation approaches limiting service delivery sites and access 3, 4. This has particularly been the case in sub‐Saharan Africa owing largely to strained national health budgets.

Epidemiological modelling suggests that PrEP will be most cost effective if offered to those at highest risk 5, 6, 7, 8, 9, and where PrEP has gained momentum both in community support and scale‐up among World Health Organization (WHO) recommended higher risk populations 10, such as Australia and the United Kingdom, significant falls in HIV incidence have been observed 11, 12. Conversely, many of the phased approaches have come with limited demand generation and communications strategies 3. These barriers perpetuate challenges for uptake and effective use of PrEP among current and potential end‐users. Rendering the UNAIDS goal of reaching three million people at high risk with PrEP by 2020 unlikely 13.

As programmes shift to integrate PrEP, it is crucial that we understand the perspectives of those taking up and using PrEP in order to demonstrate demand, facilitate use, and mitigate multi‐level barriers. At the centre of successful proven theories and practices concerning intervention scale‐up are time, communication and user‐acceptability 14. Acceptability can be defined in a myriad of ways, but the key elements, also reflected in this paper, include: (1) the applicability of the innovation (oral PrEP), or the relevance and responsiveness of the innovation to the lives of those who need it; as well as, (2) the acceptability which speaks to a more internal, emotional, user‐centred 15) perspective. Fundamentally, it can be argued that the uptake and continued use of PrEP will occur at scale only if populations/end‐users are at the centre and PrEP is: a known product, meets needs, can be integrated into everyday lives. Our previous research relating to female initiated HIV prevention technologies demonstrated how, historically, interventions have focused on the easiest goals, such as ensuring clinical access and examining product attributes 16. However, it was often personal and relationship concerns, such as comfort, trust, and sexual pleasure, in addition to practicalities, that actually played a central role in uptake and use 16. As the rollout of PrEP continues, it is essential that researchers and implementers continue to examine how it is influencing and changing the lives of end‐users.

In this review, we synthesize published literature concerning both the actual experience of using PrEP and the perspectives of potential end‐users (which we refer to as “theoretical use”). This work builds on previous mixed method and qualitative reviews 16, 17. Our aim is to elucidate the broad perception or experience of PrEP, including motivations for use, barriers to uptake or continued use, and the manner in which it impacts (or could impact) everyday life. We seek to highlight current gaps in the literature to facilitate the scale‐up of PrEP.

## 2 Methods

This research utilizes scoping review methods to establish the extent, range, and nature of published literature assessing the experience of PrEP uptake and use, or the theoretical perspective of use, in terms of applicability and acceptability 18. Often, but not always, a forerunner to full systematic reviews, scoping reviews provide a means of rapidly appraising emerging subject matter and can provide a mechanism by which complex findings are summarized for policy makers and practitioners. Arksey and O'Malley outline five necessary phases: identification of the research question; identification of relevant studies; study selection; charting the data; and collation, which summarizes and reports results 18.

Our review of the literature was informed by the question, “What are the lived experiences or personal perspectives of those HIV at‐risk populations in sub‐Saharan Africa adopting and using PrEP?” Acknowledging the growing interest and motivation to expand review methods to allow for less rigid, and more inclusive, synthesis of data 19, we sought to combine data from mixed methods research (i.e. both quantitative and qualitative) as well as lessons learned from pre‐cursor studies which addressed theoretical rather than actual acceptability and use of PrEP. We also sought to look across HIV at‐risk populations to explore where there may be commonalities or differences in perspectives on PrEP.

The search was conducted in August 2018 in PubMed and PsycInfo using the keywords: Africa (and all countries classified in the UN region), pre‐exposure prophylaxis (PrEP), Acceptability, Willingness, Barriers, Facilitators, Use and Preferences. Papers were included if they: focused on theoretical or actual use of PrEP and took place in Africa. After deduplication, the search returned 68 papers, which were divided among all three authors for review. Thirty‐three were excluded for the following reasons: they had no bearing on the topic; did not include primary, empirical data (e.g. was a review or a study protocol); contained only research relating to providers, policy maker or other perspectives on PrEP; or research took place outside of Africa. There were no date or language exclusions. Note that studies only emerged from sub‐Saharan Africa.

All three authors extracted and compiled relevant data in a spreadsheet. Bibliographic data, study populations and study locations, as well as whether the study involved actual use or theoretical perspectives of PrEP (e.g. “If PrEP were to be available in your area, would you wish to use it?” and associated data) were recorded in addition to themes covered in the papers. Once all data were compiled, we conducted a broad content analysis 20. Codes, concepts, and ideas were documented, before being organized into relevant meta‐themes. Authors then reviewed the consolidated dataset of all data and author findings. A limited subset of the final list of included papers was reviewed by two of the authors to ensure agreement of findings. This analysis also built upon a previous adapted meta‐ethnography (led by two of the authors of this paper), which developed a framework for understanding user‐perspectives of female initiated biomedical HIV prevention products 16. Note that where a mixed‐methods paper was included, the quantitative data were incorporated to support the qualitative themes (e.g. such as perspectives on risk compensation and statistical analyses showing none was found). Additionally, there is no quality assessment of data since this review is focused on exploring data to elucidate current understanding of PrEP and identify areas for further research. Finally, since the results and discussion are combined in this paper, additional supporting reviews and data papers are included in the sections that follow.

## 3 Results and discussion

This review generated 35 papers primarily from studies of potential but not actual PrEP users, from qualitative components of product efficacy studies, or from participants in demonstration projects undertaken in sub‐Saharan Africa, where HIV infection remains both common and very highly stigmatized. These studies include a range of populations including male and female sex workers; men who have sex with men (MSM); HIV serodiscordant couples; adolescent girls and young women (AGYW); pregnant and breastfeeding women; women partners of migrant workers; and people who use drugs (PWUD). Included papers are listed in Table 1.

**Table 1:** Review papers included

Authors	Title	Pub year	Type of PrEP use (theoretical/actual)	Population(s)	Location
Agot *et al*.	Accuracy of self‐report and pill count measures of adherence in the FEM‐PrEP clinical trial: implication for future HIV‐prevention trials	2015	Actual	High‐risk adult women	Kenya, South Africa, Tanzania
Amico *et al*.	Experiences with HPTN 067/ADAPT study‐provided open‐label PrEP among women in Cape Town: facilitators and barriers within a mutuality framework	2017	Actual	High‐risk adult women	South Africa
Bazzi *et al*.	Perspectives on biomedical HIV prevention options among women who inject drugs in Kenya	2018	Theoretical	Women who inject drugs	Kenya
Busisiwe *et al*.	Influences on visit retention in clinical trials: insights from qualitative research during the VOICE trial in Johannesburg, South Africa	2014	Actual	High‐risk adult women	South Africa
Carroll *et al*.	Gendered differences in the perceived risks and benefits of oral PrEP among HIV serodiscordant couples in Kenya	2016	Actual	Serodiscordant couples	Kenya, Uganda
Corneli *et al*.	Motivations for reducing other HIV risk‐reduction practices if taking pre‐exposure prophylaxis: findings from a qualitative study among women in Kenya and South Africa	2015	Theoretical	High‐risk adult women	Kenya, South Africa
Corneli *et al*.	Facilitators of adherence to the study pill in the FEM‐PrEP clinical trial	2015	Theoretical	High‐risk adult women	Kenya, South Africa
Corneli *et al*.	Participants’ explanation for nonadherence in the FEM‐PrEP trial	2016	Actual	High‐risk adult women	Kenya, South Africa
Corneli *et al*.	A descriptive analysis of perceptions of HIV risk and worry about acquiring HIV among FEM‐PrEP participants who seroconverted in Bondo, Kenya and Pretoria, South Africa	2014	Actual	High‐risk adult women	Kenya, South Africa
Corneli *et al*.	The science of being a study participant: FEM‐PrEP participants’ explanations for overreporting adherence to the study pills and for the whereabouts of unused pills	2015	Actual	High‐risk adult women	Kenya, South Africa
Eakle *et al*.	Exploring acceptability of oral PrEP prior to implementation among female sex workers in South Africa	2018	Theoretical	FSW	South Africa
Luecke *et al*.	Stated product formulation preferences for HIV pre‐exposure prophylaxis among women in the VOICE‐D (MTN‐003D) study	2016	Actual and theoretical	High‐risk adult women	South Africa
Fowler *et al*.	Attitudes of serodiscordant couples towards antiretroviral‐based HIV prevention strategies in Kenya: a qualitative study	2014	Theoretical	Serodiscordant couples	Kenya
Kibengo *et al*.	Safety, adherence and acceptability of intermittent tenofovir/emtricitabine as HIV pre‐exposure prophylaxis (PrEP) among HIV‐uninfected Ugandan volunteers living in HIV‐serodiscordant relationships: a randomized, clinical trial	2013	Actual	Serodiscordant couples	Uganda
Mutua *et al*.	Safety and adherence to intermittent pre‐exposure prophylaxis (PrEP) for HIV‐1 in African men who have sex with men and female sex workers	2012	Actual	MSM, FSW	Kenya
Guest *et al*.	Acceptability of PrEP for HIV Prevention Among Women at High Risk for HIV	2010	Actual	High risk adult women	Ghana
Falcao *et al*.	Willingness to use short‐term oral pre‐exposure prophylaxis (PrEP) by migrant miners and female partners of migrant miners in Mozambique	2017	Actual	Male migrant miners and female partners	Mozambique
Hartmann *et al*.	Motivated Reasoning and HIV Risk? Views on Relationships, Trust, and Risk from Young Women in Cape Town, South Africa, and Implications for Oral PrEP	2018	Theoretical	Young women	South Africa
Namey *et al*.	When and why women might suspend PrEP use according to perceived seasons of risk: implications for PrEP‐specific risk‐reduction counselling.	2016	Theoretical	Sexually active women at higher risk of HIV	Kenya, South Africa
Mack *et al*.	The importance of choice in the rollout of ARV‐based prevention to user groups in Kenya and South Africa: a qualitative study	2014	Theoretical	FSW, Serodiscordant couples, AGYW,	Kenya, South Africa
Ngure *et al*.	I Knew I Would Be Safer. Experiences of Kenyan HIV Serodiscordant Couples Soon After Pre‐Exposure Prophylaxis (PrEP) Initiation	2016	Actual	Serodiscordant couples	Kenya
Mugo *et al*.	Understanding Adherence to Daily and Intermittent Regimens of Oral HIV Pre‐exposure Prophylaxis Among Men Who Have Sex with Men in Kenya	2015	Actual	MSM	Kenya
Pintye *et al*.	HIV‐Uninfected Kenyan Adolescent and Young Women Share Perspectives on Using Preexposure Prophylaxis During Pregnancy	2018	Theoretical	Young pregnant or post‐partum women	Kenya
Pintye *et al*.	“I Did Not Want to Give Birth to a Child Who has HIV”: Experiences Using PrEP During Pregnancy Among HIV‐Uninfected Kenyan Women in HIV‐Serodiscordant Couples	2017	Actual	Serodiscordant couples	Kenya
Restar *et al*.	Perspectives on HIV Pre‐ and Post‐Exposure Prophylaxes (PrEP and PEP) Among Female and Male Sex Workers in Mombasa, Kenya: Implications for Integrating Biomedical Prevention into Sexual Health Services	2017	Theoretical	Young male and female sex workers	Kenya
Roberts *et al*.	Intimate Partner Violence and Adherence to HIV Pre‐exposure Prophylaxis (PrEP) in African Women in HIV Serodiscordant Relationships: A Prospective Cohort Study	2016	Actual	Negative women in serodiscordant relationships	Kenya, Uganda
Robinson *et al*.	“How I Wish This Thing Was Initiated 100 Years Ago!” Willingness to Take Daily Oral Pre‐Exposure Prophylaxis among Men Who Have Sex with Men in Kenya	2016	Theoretical	MSM	Kenya
Shaver *et al*.	Comparing Provider and Client Preferences for HIV Prevention Services in South Africa among Men Who Have Sex with Men	2017	Theoretical and actual	MSM	South Africa
Sithole *et al*.	HIV prevention needs for men who have sex with men in Swaziland	2017	Theoretical	MSM	Swaziland
Van der Elst *et al*.	High acceptability of HIV pre‐exposure prophylaxis but challenges in adherence and use: qualitative insights from a phase I trial of intermittent and daily PrEP in at‐risk populations in Kenya	2012	Actual	MSM, FSW	Kenya
van der Straten *et al*.	Perspectives on use of oral and vaginal antiretrovirals for HIV prevention: the VOICE‐C qualitative study in Johannesburg, South Africa	2014	Actual	High‐risk adult women	South Africa, Uganda, Zimbabwe
van der Straten *et al*.	Women's experiences with oral and vaginal pre‐exposure prophylaxis: the VOICE‐C qualitative study in Johannesburg, South Africa	2014	Actual	High‐risk adult women	South africa, Uganda, Zimbabwe
Ware *et al*.	Lay Social Resources for Support of Adherence to Antiretroviral Prophylaxis for HIV Prevention Among Serodiscordant Couples in sub‐Saharan Africa: A Qualitative Study	2015	Actual	Serodiscordant couples	Uganda
Ware *et al*.	Integrated delivery of antiretroviral treatment and pre‐exposure prophylaxis to HIV‐1 serodiscordant couples in East Africa: a qualitative evaluation study in Uganda	2018	Actual	Serodiscordant couples	Uganda
Ware *et al*.	What's love got to do with it? Explaining adherence to oral antiretroviral pre‐exposure prophylaxis for HIV‐serodiscordant couples	2012	Actual	Serodiscordant couples	Uganda

Very few studies reported clear opposition to PrEP as an HIV prevention tool, although some generated evidence of community distrust of study/trial designs or of the concept of PrEP itself 21. Theoretical studies among potential end‐users typically found high acceptability of PrEP 22, 23, 24, 25, 26, 27, 28, 29, 30, 31. By including the theoretical research, a range of regimens are included in the review including: oral daily and intermittent (the definition of which is particular to the given study), as well as emerging injectable and vaginal ring products.

One theoretical study suggested a limited motivation to take PrEP in light of HIV treatment needs and few pre‐existing social norms relating to prophylactic medication 24. However, no clinical trial or demonstration project reported significant challenges with recruitment, potentially due to financially incentivized participation and/or provision of higher calibre health care as compared to the norm 32. Some studies reported altruism 32 or “Ubuntu,” 21 the concept of contributing something positive to your community, as research motivators to advance HIV prevention. Actual use research also found PrEP acceptability to be high but also described a range of benefits and problems that can arise, which we address under five key themes below.

### 3.1 Balancing the complexities of personal empowerment and potential stigma

The potential to use anti‐retroviral therapy (ART) to prevent HIV infection seemed counter‐intuitive to some potential users where prior experience was focused on treating sick people with ART. When PrEP was described to a naïve potential user, concern was common that taking the same medications used to treat diagnosed HIV will mean “people will just assume I have HIV” 23. Such findings suggest that HIV related stigma is still so pervasive that it may pose a challenge to PrEP provision whether or not it is linked to services also providing ART to HIV positive people 33.

Several studies suggest that PrEP is conflated with ART not just in personal understandings of PrEP but in expectations of how others will respond, generating fear of HIV‐related stigma and discrimination. This potential stigma has been highlighted among MSM in Kenya 23; women who inject drugs in Kenya 30; female sex workers in Kenya and South Africa 34, 35; female partners of migrant workers returning to Mozambique for short periods 36; and women at high risk of HIV infection in South Africa 37, 38, 39. Participants in all these studies broadly welcomed the opportunity to avoid HIV infection but were apprehensive about being seen to take ART. All feared being identified as HIV infected which would lead to social isolation and other harms. Some went to great lengths to disguise their involvement in research and were secretive about using PrEP with regular partners and immediate family, even when this made adherence very difficult. These fears could ultimately affect the impact of PrEP where related use becomes limited.

Conversely, PrEP use in some studies raised hope and offered potentially transformative opportunities. Participants cited increased control over one's sexual health 40 and hope in avoiding infection as values of taking PrEP, especially among women with limited trust in the monogamy of their partner and limited power to ensure condom use. This is also true for men and women involved in selling sex or in known serodiscordant relationships. By offering negative partners of people with diagnosed HIV a semblance of control, it provided a means to sustain a desired relationship and/or to have children 41. Similarly, male and female sex workers in Kenya saw PrEP as a potential “expression of self‐love and self‐care” tantamount to “making a choice to live” 27.

### 3.2 Navigating the risk environment: perceptions, realities and compensation

PrEP has the potential to slow HIV transmission in geographic areas, or in sub‐populations, where there remains high HIV prevalence with low sustained viral suppression, and otherwise few viable options for prevention. Compared to condoms it is especially useful for those populations with limited control over their risk of sexual exposure (as is sometimes the case for sex workers), or others where there is a sustained risk of infection is sustained, such as people in serodiscordant relationships.

For women and girls in patriarchal societies, PrEP may be the first viable HIV prevention option that they can use on their own terms. In situations where they struggled to control exposure to HIV from male partners, and where sex outside the relationship was a concern, access to PrEP was welcomed 29, both as a means to manage risk perception and to avoid actual infection. Even within marriage and during pregnancy, women worried partners might “bring HIV into the home” 36, 41. Many women only acknowledged risk of HIV infection within their primary relationship 31 but fear of rape and other violence was also cited as a motivator for PrEP use 25.

In two earlier theoretical studies, concerns about PrEP “replacing” or decreasing condom use in general were common among sex workers 22, 34. However, male and female sex workers overall were supportive of the idea of PrEP providing “added protection” even in the context of aspirations to always use condoms 27. Additionally, three other papers noted that risk behaviour did not change over time among participants actually using PrEP including migrant workers and female partners, MSM, FSW and serodiscordant couples 36, 42, 43. It should also be noted that two systematic reviews also have shown no significant changes in behaviour among PrEP users 1, 17.

### 3.3 Relationship influences and expectations

Oral PrEP related research has documented how partners of potential or actual users have significant influence over use, as has been the case with other products. Condom use, or lack thereof, is often determined by male partners 44, 45, 46, 47, which was similarly found in microbicide gel studies^37^, ^38^. Indeed, in a theoretical study about motivations to use PrEP 32 female participants believed that the tablet would help to alleviate challenges they faced in partner condom negotiation. They described how condoms were a source of conflict in relationships where men insisted against use and women held genuine concerns about the risk of acquiring HIV or other STIs where a partner's status was unknown or the partner was suspected of external relationships.

While some have sought to promote antiretroviral (ARV) based prevention as a female controlled or initiated HIV prevention technology 48, 49, several studies have shown that male partners still often exert considerable influence, either positive or negative. Carroll *et al*. examined gender dynamics within relationships and how these influence decision making relating to PrEP, identifying a wide variety of experiences 50. HIV‐negative women in their study overwhelmingly reported that the decision for either partner to initiate ART or PrEP, as appropriate, belonged entirely to their husbands. HIV‐positive men also reported that they possess the ultimate authority to make medical decisions for themselves and their spouses. Similarly, HIV‐negative men expressed frustration with the PrEP regimen and indicated that the burden of taking medication had been thrust upon them by their HIV‐positive wives 50.

In Roberts *et al*., 16% of women in their serodiscordant couple study reported intimate partner violence (IPV) at some point during the trial 51. They also had increased risk of low adherence as assessed by pill count and by plasma tenofovir. Verbal, economic and physical IPV were all associated with low adherence. In‐depth interviews identified several ways in which IPV affected adherence, including stress and forgetting, leaving home without pills, and partners throwing pills away. Conversely, in a theoretical study, female sex workers articulated fear of violence as a motivator for taking PrEP 34.

Despite these issues, women commonly cited the option of disclosure as a benefit of PrEP use 16, 22, 34, 52, 53, thus empowering to prevent HIV infection in scenarios where their partners insist on condomless sex. Indeed, in the Partners Demonstration Project, PrEP provided additional protection as a “back‐up” mechanism when their partners refused to use condoms and in cases of condom breakage 54. However, other papers identified a wide range of disclosure decisions and experiences. In their study of PrEP adherence influencing factors as part of FEM‐PrEP, Corneli *et al*. reported some male partners of female participants were very supportive and would remind them to take the pills 31. Some had partners who merely acquiesced to their pill use, while a few opted not to tell their partners at all for fears of negative reaction and insistence of cessation.

Ware *et al*. report that while the presence of HIV serodiscordance can destabilize a couple, given that the HIV‐negative partner often reacts with anger or fear to the HIV infection (and the potential infidelity it represents), PrEP can be seen as a solution 55. Ultimately it can provide a means of safeguarding health without ending the relationship. Simultaneous use of ARVs by the HIV‐positive partner turned management of HIV into a shared experience, and serodiscordant couple‐focused services (including attending appointments and counselling as a couple) brought partners together, increasing mutual support through having a space to become educated and comfortable with PrEP 41, 56. This might suggest a potential positive impact of PrEP in developing relationship intimacy, a key aspect of health and well‐being that is often overlooked in public health and social care interventions 57.

Several papers briefly highlighted the complexities of PrEP monitoring and adherence for people in romantic or regular relationships. They describe how adherence to PrEP may at times diminish as a consequence of changing sex patterns within the relationship, such as reduced libido as a result of partner absence from the home 29, 58. Some study participants reported they may wish to cease PrEP use if in a committed relationship, within which their concern about HIV transmission would diminish 55. This should of course be an empowered choice for all wishing to start, or stop, PrEP, but poses challenges for those seeking to utilize stringent measures of PrEP adherence that do not reflect the reality of relationships.

### 3.4 Efficacy and side effects managing PrEP use

Defining and understanding the concept of ARVs for preventing HIV is complicated and multi‐dimensional, including the management of potential stigma and understanding efficacy. For instance, some studies identified that developing an initial understanding of taking ARVs to prevent HIV remained confusing to study participants even after they had completed a rigorous informed consent process and had been participating in the study 21, 37. This emerged as a central issue the trials showing no efficacy, suggesting that misunderstanding of PrEP as an efficacious prevention modality may be a significant barrier in scale‐up.

Perceived efficacy (i.e. the extent to which PrEP prevents HIV acquisition) was found to be a significant component of acceptability among most populations 22, 35. Beliefs about PrEP efficacy were directly related to whether people could be sufficiently adherent to the medication, and were influenced by lack of communication or open support from places of authority (clinics, providers and Ministries of Health) 21. In efficacy research, the possibility of being on placebo and the unknown efficacy of the active PrEP pill were directly linked to lack of use in some studies, pointing to the importance of highlighting the high efficacy of PrEP in actual service delivery 37, 58, 59. It will be important to capitalize; however, on the curiosity about PrEP, its novelty and its protective value if taken consistently, to encourage use among those at highest risk 23, 54.

Side effects, or fear of potential side effects, were common in both efficacy and implementation studies. Initial side effects among female partners of migrant mine workers in Mozambique dissipated over time but were tolerated because they took the side effects to mean that PrEP was working 36. Concerns about side effects during discussions of theoretical acceptability were common among women in one study, including partners in serodiscordant couples, adolescent girls and young women (AGYW), and sex workers 22. Sex workers in Kenya suggested they would prefer intermittent use to reduce potential side effects over time since they anticipated a longer period of potential exposure to HIV as a result of their work 27. This should also be noted as a potential motivator for intermittent PrEP use.

PrEP could play an important role in safer conception, but side effects need to be clearly discussed with clients. In one study, women said they would stop PrEP if trying to conceive due to worry of how it might affect the foetus 29. However, women in the Partners study in Kenya who experienced minor side effects still affirmed that having an HIV‐negative baby was worth the risk 41.

Finally, it should be noted that a recent meta‐analysis suggested that very few major, and limited minor, side effects will occur when using PrEP 1. This has been confirmed by multiple demonstration projects where minor side effects were limited to the first few days/weeks of use 60. This suggests a significant disjuncture between the perception of potential side effects, the attribution of illness to the use of PrEP, and side effects actually triggered by the medication. Communicating this to interested, but concerned, end‐users should be a priority for intervention scale‐up.

### 3.5 Practical considerations for PrEP use

User preferences for specific product attributes and the practicalities of PrEP use have been widely investigated 16. These practical considerations remain important in planning for PrEP scale‐up both for service delivery and day‐to‐day end‐user experiences. Among actual end‐users, basic practicalities such as clinic access, pill storage and managing adherence in the context of everyday obligations were significant considerations, as was negotiating with family, schools or employers to make time for appointments 23, 33.

The service delivery environment also played a key role in successful PrEP implementation. This was a particularly pressing issue for key populations who already commonly face stigma attending health clinics 61. Fear of rebuke from providers for not adhering to the medication, or being dropped from a study as a result, was cited as reason for lying about adherence 62, 63, and points to the need to ensure constructive, open engagement between providers and clients. Open exchanges and high quality counselling were noted as being of particular value 32. Negative clinic experiences directly deterred PrEP use 21, 33, whereas good quality care was highly valued and increased clinic attendance 33, 58. In the FEM‐PrEP study, repeated HIV‐negative tests (even if on placebo) reinforced the notion that PrEP worked 32, 33, although in another study it was noted that having to test often for HIV could be a barrier to continued PrEP use 36.

In Kenya and Uganda, women felt that providers play a key role in supporting PrEP use, especially outside of the trial environment for women who may not fully understand their own risk and the benefits of PrEP for conceiving babies free of HIV 41. Women in Uganda also worried that the shortage of doctors/clinical staff could stand in the way of PrEP rollout due to already limited capacity to deliver existing services 59. Importantly, community awareness of PrEP to promote support for use, access, and coverage has been noted as a critical component for successful implementation 26, 34, 41.

Consistent adherence while taking PrEP is the cornerstone of effective use and yet it was challenging in several of the efficacy studies to maintain adequate levels of use 1. Women in several studies acknowledged forgetting, ambivalence, personal barriers (travel, family issues), missing clinic visits and refills, and worry about side effects as reasons for inconsistent use 37, 43, 58, 62. Similarly, the active management of actual side effects is an important factor to consider in scale‐up, directly linking with the need for supportive counselling to encourage consistent use over time. Conflicts with partners and having to hide pill taking was also reported to cause people to forget to take their pills 58.

The use of alcohol and drugs was another concern related to efficacy and consistent use. This was particularly highlighted among sex workers, MSM, and young women, where participants were concerned that PrEP may not protect them from HIV when using other substances 22, 23, 34. These participants also acknowledged potentially forgetting to take their pills if they were inebriated. Some potential PrEP users reported their own prior inability to finish pills (such as antibiotics) as a signal they might not be able to successfully take oral PrEP 23.

Overall, the importance of developing pill taking behaviours and strategies is paramount to successful use and most have found manageable solutions 21, 36. Behaviours can be supported by early, and continued engagement with counsellors which has been shown to generate consistent use 54. Interestingly, one older study found that those experiencing more difficulties in pill taking were more likely to stay in the study and were also able to develop strategies over time 64. Related to the supportive environment and intimate relationships, studies that involved serodiscordant couples found the relationship provided an easier context in which to use PrEP, as it became a “shared commitment to HIV management and the relationship” 54 where adherence/use was considered a “family affair” 59.

Diverse perspectives were shared in relation to alternative, non‐daily PrEP options. For some populations, such as female partners of migrant miners 36, shorter term use was preferable and intermittent dosing, for when partners were at home, was appealing.

Some of the papers in this review included theoretical perspectives on future products where women expressed preferences for long‐acting PrEP 34, 52. One paper reported how some women anticipated their preferences would change over time 39, and some participants expressed wanting to have systemic protection while others prefer to have drug only in one area of their bodies 39. Highlighting the importance of choice in this VOICE‐D ancillary study South Africa, young women preferred pills which seemed less dangerous and more trustworthy, while female partners in serodiscordant couples and sex workers preferred long‐acting products which require less maintenance 22. These perspectives will be important to consider within the context of all that has been learned through oral PrEP implementation to ensure access to a range of appropriate and relevant interventions and products as they emerge.

## 4 Conclusions

This review demonstrates the ways in which PrEP can, and already is, having a significant impact on the personal, relational, and social lives of HIV affected populations in sub‐Saharan Africa. Those considering investment in and implementation of PrEP should take note of the robust and nuanced evidence suggesting great potential for PrEP to reduce HIV related anxiety, empower people (especially women) to take control of their sexual health, and to positively influence relationships (particularly those that are serodiscordant). These are significant benefits playing a role in reducing new infections.

However, this review also makes clear that the incorporation of PrEP into everyday lives is not without its challenges. There are pervasive concerns about side effects, and, to some extent, that the current required clinical engagement is overly burdensome. These issues exist in combination with health services that can be stigmatizing or insufficiently welcoming of key populations 65. Uncertainty as to the effectiveness of PrEP remains especially where ingrained messages featuring 100% condom use has been the focus of HIV prevention campaigns over several decades. Belief and trust in PrEP will take time to develop and achieving this will also require the maintenance of efforts to reduce HIV‐related stigma. Several studies identified how conflation of PrEP as ART and therefore being HIV positive could be perceived negatively by partners, family or friends. Through PrEP education and addressing HIV related stigma we can reduce this concern and potential barrier.

Notions of risk compensation were not common among the actual use studies in this review, and reported sexual behaviour did not change over time in any of the efficacy trials or demonstration projects 1, 66, 67. However, this has continued to be a concern expressed from higher level stakeholders. Rhetoric perpetuating the assumption that PrEP will automatically encourage increased risk‐taking behaviours should be carefully monitored to avoid.

Much of the research to date has emerged within the confines of clinical trials or demonstration projects. As such, some of the findings reported may be specific to that environment and may not have (as much) relevance in real‐world settings. Examples include positive feelings towards PrEP in the context of high quality wrap around study services; uncertainties about efficacy of drugs given where randomization to placebo was possible; and practical concerns of being seen attending a trial site often synonymous with HIV treatment. Such issues may be unavoidable in the shorter‐term given the requirement for rigorous HIV testing but it will be important to mitigate these issues in longer‐term implementation.

Thus far research has been perhaps overly focused on practical dimensions of adherence (i.e. where pills are kept, how they are stored etc.). Less often have papers examined how significant others shape and inform continued PrEP use, especially the ways in which couples could be supported to effectively negotiate use within their relationship. It is interesting to note that despite much global level discourse framing PrEP as emancipatory for women – they finally have an efficacious female initiated technology to protect themselves from HIV ‐ it seems there is good evidence that many men still wield significant authority over if and how it is used. Given socially and culturally pervasive gender imbalances, this should not be surprising, and is a reminder to be cognisant of how the notion of empowerment is communicated.

Notable in their (near complete) absence from this review are people who use drugs and transgender people. Only one paper specifically examined the theoretical perspectives of people who use drugs, and in brief, reports only on a concern for side effects. Marginally more attention has been paid to MSM but not the extent that is required given high HIV incidence across the continent 65. Further research with MSM, PWUD and transgender people is required to understand issues such as relational support for PrEP use, stigma that may be associated with use and how to address it, as well as the effects of criminalization on access to PrEP and continued PrEP use. Criminalization of key populations and practices such as sex work was not specifically addressed in the papers identified in this review and should be carefully considered as a significant barrier to PrEP use. Papers outside this review have also underscored this point 68, 69.

This paper describes a scoping review of available research relating to the experience of using PrEP, or perspectives about potentially doing so. It presents the extent of evidence and summarizes key issues that may influence uptake and continued use by key populations. It is not, however, a systematic review or meta‐analysis of all available data. Our review was limited to peer reviewed academic articles and does not include grey literature. This is a rapidly emerging field of research and many studies of real‐world PrEP use are still being analysed and disseminated. The paper does, however, provide a comprehensive overview of significant issues that have relevance to demand creation, health promotion, and clinical interventions that aim to increase uptake and effective use of PrEP.

## Competing interests

The authors have no competing interests to declare.

## Authors’ contributions

R.E. conceived the paper. A.B. and R.E. conducted the initial literature search, and all authors extracted and reviewed data, compared findings, and developed thematic categories. R.E., A.B. and P.W. co‐wrote the paper and approved the final version.
